# Immunostimulatory Polymers as Adjuvants, Immunotherapies, and Delivery Systems

**DOI:** 10.1021/acs.macromol.2c00854

**Published:** 2022-08-04

**Authors:** Adam M. Weiss, Samir Hossainy, Stuart J. Rowan, Jeffrey A. Hubbell, Aaron P. Esser-Kahn

**Affiliations:** † Pritzker School of Molecular Engineering, University of Chicago 5640 S. Ellis Ave., Chicago, Illinois 60637, United States; ‡ Department of Chemistry, University of Chicago 5735 S Ellis Ave., Chicago, Illinois 60637, United States

## Abstract

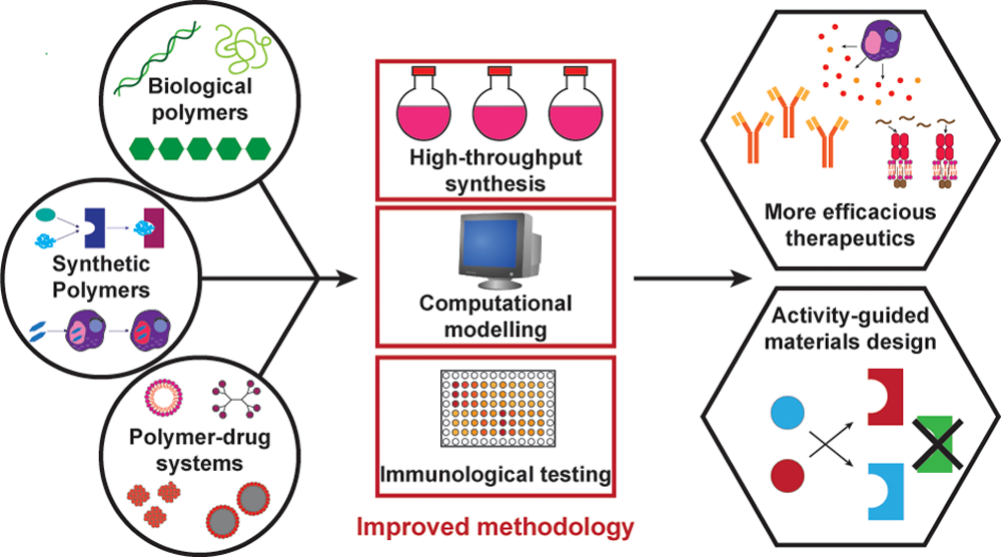

Activating innate immunity in a controlled manner is necessary for the development of next-generation therapeutics. Adjuvants, or molecules that modulate the immune response, are critical components of vaccines and immunotherapies. While small molecules and biologics dominate the adjuvant market, emerging evidence supports the use of immunostimulatory polymers in therapeutics. Such polymers can stabilize and deliver cargo while stimulating the immune system by functioning as pattern recognition receptor (PRR) agonists. At the same time, in designing polymers that engage the immune system, it is important to consider any unintended initiation of an immune response that results in adverse immune-related events. Here, we highlight biologically derived and synthetic polymer scaffolds, as well as polymer–adjuvant systems and stimuli-responsive polymers loaded with adjuvants, that can invoke an immune response. We present synthetic considerations for the design of such immunostimulatory polymers, outline methods to target their delivery, and discuss their application in therapeutics. Finally, we conclude with our opinions on the design of next-generation immunostimulatory polymers, new applications of immunostimulatory polymers, and the development of improved preclinical immunocompatibility tests for new polymers.

## 1 Introduction

Polymers hold immense potential for the development of novel therapeutics. On account of their high molecular weights, tunable properties, and ability to assemble into ordered nano- and microstructures, polymers can deliver molecular cargo, form interactions with biological molecules, and target specific cell subsets, making them potent tools for treatment of disease or modulation of biological systems.^1−3^ While such applications of polymers are commonly exploited, it has also become apparent that polymers hold an innate immunostimulatory capacity, which can result in enhanced immune responses or toxic side-effects when applied in therapeutic modalities.^4^ Indeed, nature’s polymers, such as bacterial peptidoglycans and single-stranded DNA, are potent immunogens that are critical for immune recognition of “non-self” or “damaged-self” from “self” (e.g., recognizing viral or cancerous proteins from endogenous proteins).^5−7^ Polymeric adjuvants can enhance cellular uptake,^8^ bind immune receptors with higher affinity and avidity,^9^ and alter pharmacokinetics^10^ relative to small molecule adjuvants. Synthetic alternatives to nature’s polymers offer a low-cost and tunable parameter space for the design of new adjuvants and delivery systems that can be prepared with optimized biological responses.

The premise that polymers can modulate innate and adaptive immune responses has a remarkably long precedent. In the 1930s, Goebel and Avery reported several landmark studies demonstrating that conjugation of carbohydrate polymers to proteins could modulate the immune response in a pneumococcus vaccine.^11,12^ Later, researchers in the 1960s identified that hydrophilic polymers such as poly(ethylene glycol), alginates, and methylcelluloses were safe for use as drug excipients or surgical tools, while other hydrocarbons such as polystyrene and poly(vinyl chloride) were less favorable for biological applications.^13,14^ Similar property–activity relationships were developed through the 1980s and provide a foundational understanding of polymer biocompatibility today. Work in the 1990s demonstrated that polymers could be synthesized with precise chemistries to deliver small molecules, proteins, or oligonucleotides with controlled release kinetics, biodistribution, and immune responses.^15−20^ Today, the ascent of controlled polymerization techniques combined with sophisticated monomer design^21^ has allowed a breadth of polymers to be used in biomedical applications such as tissue scaffolds, drug delivery systems, drug excipients, antimicrobial coatings, and gene therapies, to name a few. Specific to clinical immunology, polymers are critical components of liposomal and nanoparticulate vaccine formulations,^1,4,22^ transfection reagents for CAR T cell production and oncological gene therapies,^4,17,23^ and compatibilizing agents for stents and devices.^4^ Growth in the use of polymers in these applications requires an increased molecular understanding of interactions between polymers and the immune system to allow for the development of safe and improved therapeutics.

In this Perspective, we discuss the current status of immunostimulatory polymers for therapeutic applications. While polymers employed for drug delivery and nanomedicine have been extensively reviewed,^2,24,25^ herein we discuss the immune response directed toward polymers independent of cargo or other extrinsic stimuli. Rather than focus on specific disease contexts, we instead discuss design principles necessary for directing an immune response using one of four classes of materials: (1) biologically derived (or biologically inspired) polymers that bind known pattern recognition receptors (PRRs, described below), (2) synthetic polymers that bind known PRRs, (3) polymers that are covalently conjugated to or noncovalently formulated with PRR ligands to enhance adjuvanticity, and (4) polymers that can direct the delivery of PRR ligands to specific immune compartments using stimulus-responsive chemistry, biodegradable functional groups, or targeting ligands. We then discuss how increased understanding of immune receptor signaling, machine learning, and computation-guided design, high-throughput synthesis and screening, and other strategies will allow for the design of materials with a higher capacity for PRR binding and adjuvanticity. Finally, we note the importance of biocompatibility screening in the development of polymeric adjuvants and propose methods by which this can be achieved.

## 2 Immune System Overview

**Figure 1:**
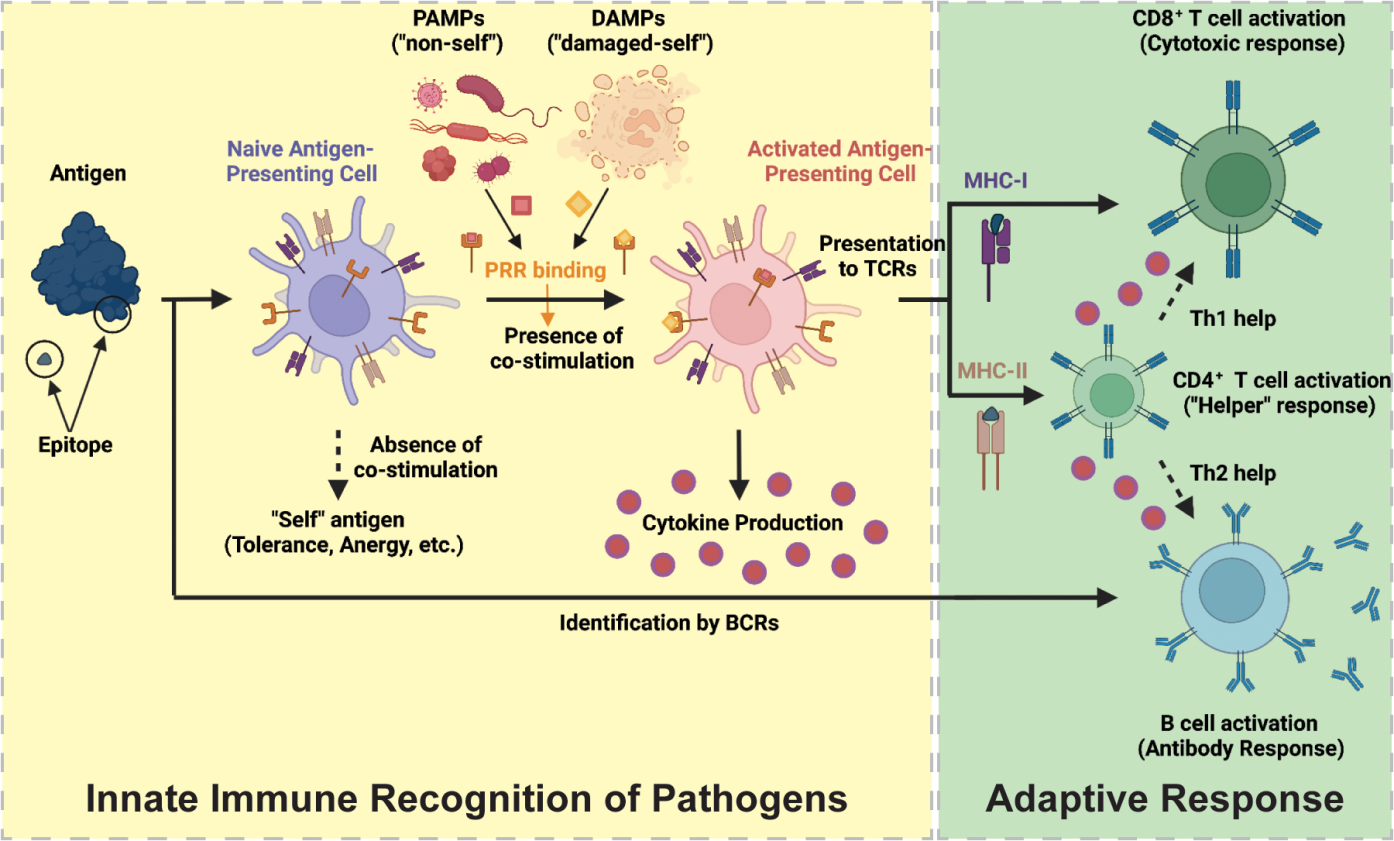
Schematic overview of relevant components of the innate and adaptive immune systems for vaccination and cancer immunotherapy. Under a disruption of homeostasis, antigen presenting cells (APCs) of the innate immune system identify “non-self” and “damaged-self” molecular motifs (PAMPs and DAMPs) through their major histocompatibility complexes (MHC-I and MHC-II) and pattern recognition receptors (PRRs). APCs then become activated, secrete immunostimulatory cytokines, and present antigens on MHC-I or MHC-II to stimulate a response by the adaptive immune system. The adaptive response is coordinated by B cells, which bind antigens with their B cell receptor (BCR), as well as CD4^+^ and CD8^+^ T cells, which bind peptide:MHC complexes with their T cell receptor (TCR). Together, T and B cells facilitate the destruction of the pathogen. Created with BioRender.com.

While the immune system cannot be fully covered in a single Perspective, we provide a brief overview of some major components of innate and adaptive immunity relevant for the design of immunostimulatory polymers and their applications for vaccination and immunotherapy (Figure 1). More information about specific aspects of immunity necessary in the design of next-generation therapeutics can be found in excellent texts and reviews.^26−30^

To protect the body from disease, foreign antigens (any proteins or carbohydrates to which an immune response is mounted) must be identified by the immune system with the appropriate costimulatory molecules to generate an immune response. Antigen presenting cells (APCs), such as dendritic cells and macrophages, sample circulating proteins as they pass through secondary lymphoid organs. These notably include the lymph nodes, where APCs sample contents of the lymph draining from peripheral tissues, and in the spleen, where APCs sample contents of the blood. Under homeostatic conditions, APCs endocytose protein antigens and enzymatically process them into short peptides (epitopes), which can be loaded onto major histocompatibility complex I or II (MHC-I and MHC-II). APCs then present these epitopes on MHC-I or MHC-II to naïve T cells in the spleen and lymph nodes. In the absence of costimulatory molecules (i.e., in the case of an endogenous or “self” antigen), such antigen presentation fails to induce a productive immune response and results in anergy, exhaustion, or immune regulation (i.e., T cell differentiation toward a regulatory phenotype). During a disruption to homeostasis, such as the presence of an infection or cancer (i.e., presence of “non-self” or “damaged self”), APCs are further stimulated by pathogen associated molecular patterns (PAMPs) and/or damage associated molecular patterns (DAMPs) binding to PRRs in the cytosol or on the APC surface. Activation of these PRRs can induce production of costimulatory factors, such as cytokines (which are immune signaling proteins), which are needed to license naïve T cells to become activated, rapidly proliferate, and facilitate a memory-inducing adaptive immune response. APCs also reside in lesser numbers in peripheral tissues, such as the skin, and sample the local environment for antigens. Under stimulation by PAMPs or DAMPs, they migrate to the secondary lymphoid organs where they can similarly initiate an adaptive response.

The adaptive component of the immune response is mediated by T cell receptors (TCRs) and B cell-bound immunoglobulins (the B cell receptor, BCR). TCRs, in coordination with CD4 and CD8, bind antigenic epitopes presented by APCs on MHC-II or MHC-I, respectively, to bridge innate and adaptive immunity. T cell responses can be separated into a CD8^+^ cytotoxic T lymphocyte (CTL) response, critical for killing and clearing pathogen-infected or tumor cells, or a CD4^+^ “helper” T cell (Th) response, important for secreting soluble immune mediators, such as cytokines, which enhance CD8^+^ T cell and B cell responses. Helper T cells can be further separated into Th1 and Th2 subtypes, where Th1 responses support CTL-mediated killing and Th2 responses support B cell maturation and differentiation. BCRs, meanwhile, bind antigenic macromolecules such as proteins and carbohydrates (or, in some cases, synthetic materials) on account of their secondary structure. Naïve B cells that bind an antigen and subsequently receive stimulation by Th2-biasing cytokines differentiate into plasma cells that can both secrete antibodies and allow for phagocytosis to mediate destruction of pathogens. Alternatively, arrayed binding of a repeating secondary structure, such as in the case of a carbohydrate or synthetic material, can induce B cell maturation and antibody production independent of T cell signaling. Stimulation of B cells with appropriate cytokines can induce isotype class switching from conventional IgM and IgD antibodies to those with increased affinity and specialized functions, such as IgG2 (which specialize in responding to bacterial capsular polysaccharides) and IgA (which specialize in responding to mucosal infections).^31^ Concurrent B and T cell responses are often necessary to neutralize pathogens.

Given the importance of innate immune costimulation in mounting a productive adaptive immune response, providing PAMPs and/or DAMPs that can bind PRRs concurrently with administration of antigen is often necessary for application in vaccination and immunotherapy.^28,29^ Such molecular agonists and other molecules, which modulate adaptive responses, are called adjuvants. While prophylactic vaccines were historically generated from attenuated or inactivated pathogens, which intrinsically contain PAMPs, new systems such as subunit and mRNA vaccines or cancer immunotherapies may lack natural immunostimulatory components and can require supplementation with adjuvants to facilitate immunogenicity. There are >20 known PRRs that bind a diverse range of molecular patterns, and the design of synthetic PRR agonists that induce specific cytokine profiles for use in therapeutics is an active area of research (Table 1). Despite promising research in these areas, synthetic adjuvants are often limited by systemic toxicity, as activation of PRRs can result in immunotoxic cytokine production and lead to fever, injection site pain, or other side effects. In severe cases, excessive PRR stimulation can result in a life-threatening pathology known as a “cytokine storm” (sometimes called Cytokine Release Syndrome, or CRS).^32,33^ As such, effective formulation of antigen and adjuvant in a delivery vehicle, such as synthetic nanoparticles, emulsions, or liposomes, to target immune cell subsets and reduce adjuvant dose is often beneficial for successful vaccine design. Alternatively, adjuvants that enhance adaptive immunity through less-specific mechanisms than PRR binding (such as general inflammation or “depot” effects) have found success in clinical applications. Specifically, aluminum salts (“alum”) were among the first adjuvants, and many FDA-approved vaccines have made use of alum or squalene emulsion-based antigen depots. The mechanism of antigen depot-based vaccines is debated but appears to invoke B and T-helper cell-mediated immunity by stabilizing antigen at the injection site, altering cell adhesion, and inducing inflammation so as to recruit and activate nearby peripheral tissue-resident APCs.^33−35^ While we will focus on adjuvants that target PRRs, the ability of polymers to form biophysical interactions with cells must be considered in defining the immunogenicity of next-generation polymeric adjuvants.

**Table 1:** Selected PRR Agonists and Regulatory Approval Status^a^

PRR	ligand class	phase III (with trial number) or approval status?
TLR1/2	lipopeptides	no
TLR2/6	lipopeptides	no
TLR3	dsRNA	no
TLR4	lipopolysaccharide	FDA approved for vaccination (AS01 formulation: Shingrix; AS04 formulation: Cervarix),^36−38^ in phase III for vaccination (AS01 formulation: NCT04319380, NCT05059301)
TLR5	bacterial flagellin	no
TLR7/8	ssRNA	in phase III for vaccination (Imiquimod: NCT04083157, NCT04143451)
TLR9	CpG ssDNA	FDA approved for vaccination (CpG-1018: Heplisav B),^39^ in phase III (CpG-1018: NCT04864561 NCT04672395)
NOD (1&2)	peptidoglycan	no
NRLP3	ion flux, membrane disruption, reactive oxygen species	FDA approved for vaccination (AS01 formulation: Shingrix; AS04 formulation: Cervarix),^36−38^ in phase III for vaccination (AS01 formulation: NCT04319380, NCT05059301; Matrix-M formulation: NCT04704830, NCT04611802, NCT04120194)
STING	cytosolic cyclic DNA	no
RIG-I	short viral dsRNA	no
DNGR-1	F-actin–myosin	no
Dectin-1	β-glucan	in phase III as a cancer immunotherapy supplement (β-glucan dietary supplement: NCT04710290)
Dectin-2	α-mannan	no
C-type lectins	mannose, fucose, GlcNAc	no
DC-SIGN	high mannose glycans	no

a Note that TLR1/2 and TLR2/6 form heterodimers during ligand binding and were therefore included as one construct. Clinical trial status was identified by searching each PRR and known agonists for active or recruiting phase III clinical trial status on https://clinicaltrials.gov. Abbreviations: TLR = toll-like receptor, NOD = nucleotide-binding oligomerization domain-containing protein, NLRP3 = NACHT, LRR, and PYD domain-containing protein 3, STING = stimulator of interferon genes, RIG = retinoic acid-inducible gene, DNGR = dendritic cell natural killer lectin group receptor, DC-SIGN = dendritic cell-specific ICAM-grabbing non-integrin.

Despite the promise of small molecule and biological adjuvants in preclinical studies, existing systems are limited by poor targeting of immune cells and systemic toxicity, and few molecular adjuvants have been approved by the FDA for prophylactic vaccination. Even among recently FDA-approved molecular adjuvants, off-target toxicity has proven to induce high grade immune-related adverse events in a significant portion of the population and is thus likely to reduce vaccine compliance.^33,36,39^ Polymeric adjuvants developed with careful early stage biocompatibility testing hold promise to overcome these problems. These materials form nanostructures that can mimic the size, shape, and biodistribution of pathogens, target and deliver cargo selectively via the incorporation of immunogenic molecular patterns, and slow the systemic release of immunostimulatory components into the bloodstream. Moreover, by use of polymers that can directly bind receptors while delivering antigenic cargo, systems with multiple capacities for immunostimulation can be achieved. Understanding the design principles needed to accomplish and control such responses will facilitate the rational design of better therapeutics.

## 3 Biologically Derived Polymers with Innate Immunostimulatory Activity

### 3.1 Conceptual Overview

The innate immune system has evolved to recognize PAMPs, molecular patterns unique to pathogens, such as bacterial lipopolysaccharide, unmethylated CpG DNA, and flagellin (Table 1). Researchers have utilized such pathogenic motifs in therapeutic formulations to modulate innate immune responses and facilitate adaptive immunity.^4,40^ Biologically derived polymers with adjuvant capabilities can be extracted directly from biosources or synthesized by chemical approaches. When used in vaccines and immunotherapies, such polymers can facilitate antigen presentation, form depots, enhance endocytosis, and bind PRRs to modulate the immune response.^5^ Moreover, repetitive carbohydrate polymer structures can induce T cell-independent B cell responses.^41,42^ Carbohydrate-based formulations are employed in FDA-approved vaccines against *Hemophilius influenza* type B, *Nisseria meningitidis*, *Salmonella typhi*, and *Streptococcus pneumoniae* and are known for inducing strong Th1-biased responses.^41^ While this discussion will focus primarily on carbohydrates, glycolipids, and their derivatives as adjuvants, carbohydrates can also be employed as an antigen to invoke potent B cell-mediated immunity; excellent reviews on carbohydrate antigen vaccines can be found elsewhere.^41,42^

In the biological milieu, carbohydrates are ubiquitous as soluble or insoluble structural and functional units in cells or as glycosylation units bound to proteins called glycans.^5^ Carbohydrate-binding protein domains are known as lectins and differentiated by their carbohydrate recognition domain (CRD). A wide array of polysaccharides have been shown in an immunological context to engage the C-type lectin class of receptors and stimulate innate immune responses.^5,43^ C-type lectins include the mannose-binding lectins (which bind mannose-, fucose-, and *N*-acetylglucosamine-terminated glycans), Dectin-1 (which binds β-glucan), DC-SIGN (which binds high-mannose glycans), and many others, although significant overlap exists in terms of CRD and lectin binding specificity.^44^ Carbohydrate adjuvants targeting C-type lectins offer a promising alternative to classical adjuvants, as they are potent, synthetically tunable, and low-toxicity immune modulators. Here, we will focus on biologically derived molecular targets of C-type lectins including mannose, fucose, β-glucan, and chitosan. Other lectins, such as galectins (which bind glycans containing *N*-acetyllactosamine) and Siglecs (which bind glycans containing sialic acid), have also been shown to play a role in innate immunity and have been reviewed elsewhere.^45,46^ In addition, glycolipids derived from diverse natural products can produce robust immunological activity through PRRs or other innate immune signaling molecules including TLR4, NLRP3, and CD1d. Perhaps the most notable glycolipid is bacterial lipopolysaccharide, which was one of the earliest identified PAMPs and has been extensively reviewed for both its role in diseases and its use as an adjuvant.^6,47^ Here, we will focus on two relevant glycolipids, saponins and α-galactosylceramides (α-GalCers), as they both have recently attracted attention as adjuvants that can generate safe innate immune stimulation with unique adaptive immune response profiles.

### 3.2 Mannose and Fucose

C-type lectins with CRDs containing the amino acid sequence GluProAsn can bind mannose, fucose, and *N*-acetylglucosamine (GlcNAc).^44,48^ These lectins can be further differentiated into soluble receptors, which bind bacterial carbohydrates and signal for their destruction via the complement pathway, and cell surface receptors, which can facilitate endocytosis of antigen upon binding.^48^ We focus here on the cell surface receptors, of which DEC-205 (CD205), DC-SIGN (CD209), and the macrophage mannose receptors (MMR, or CD206) are the most studied. Mannose is a C2 glucose epimer which enzymatically reacts to form oligomers (glycosylations) at reactive sites on the surface of many proteins.^49^ Naturally or synthetically mannosylated antigens that are capable of binding these receptors enhance targeting and activation of antigen-presenting cells to facilitate adaptive immunity when used in vaccines and immunotherapies. Early works targeting the MMR identified the immunomodulatory capacity of this approach, as work by Tan and colleagues demonstrate that mannosylated antigens were more efficiently taken up by dendritic cells and presented on MHC-II than nonmannosylated antigens.^50^ Despite the promise of naturally mannosylated antigens for vaccine formulation, expression of glycosylated antigens in yeast or mammalian cells remains challenging in many cases, posing a roadblock for broad application of these systems and driving the development of synthetic mannose alternatives for therapeutic application. Synthetic alternatives have also been explored^51−53^ and are covered in greater detail in Section 5.3.

The α(1→3) linked 6-deoxygalactose sugar, fucose, can also function as a unique immunostimulatory component and holds potential for cancer immunotherapy. Fucose-rich glycans are associated with various cancers and can serve as an epitope for antibody-mediated destruction in parallel with activation of cell surface C-type lectins.^41^ In work by Liao et al.,^54^ fucose-containing polysaccharides were isolated from Reishi mushrooms and administered as an immunotherapy against a fucose-expressing Lewis lung carcinoma. The isolates were shown to elicit antibody-dependent cytotoxicity against the tumor. IgM antibody binding was probed by using a glycan microarray and revealed high affinity for terminal fucosylations reminiscent of known tumor-associated glycans. Ultimately, the group showed an increase in B cell proliferation and slowed tumor progression when mice were treated with a fucose-enriched Reishi polysaccharide fraction to account for the observed responses.^54^ Though a novel approach to immunotherapy, fucose has not been shown to induce T cell-mediated adaptive responses and likely requires combination with conventional T cell directed immunotherapies (such as checkpoint blockade) to induce productive antitumor responses in a clinical setting.

### 3.3 β-Glucans

**Figure 2:**
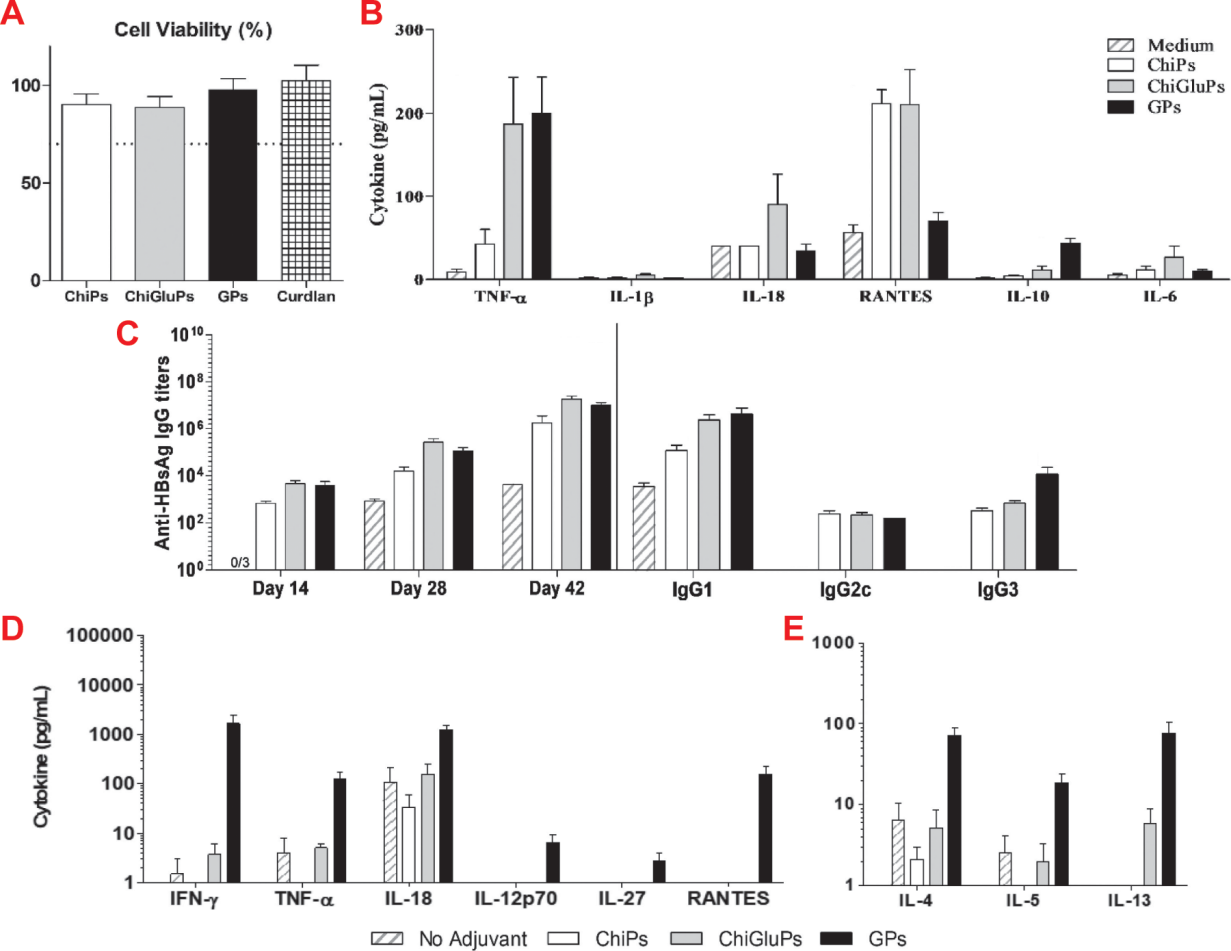
Evaluation of particulate vaccines formulated with hepatitis B surface antigen (HBsAg) and chitosan (ChiP), β-glucan derived from Curdlan (GP), or a blend of chitosan and β-glucan (ChiGluP). (A) Cell viability relative to untreated cells and (B) cytokine production of murine splenocytes treated with 200 μg/mL of the indicated formulations. (C) Antibody titers of mice. Mice were vaccinated with 400 μg/dose of the indicated formulations at days 0, 14, and 28, and total IgG titers were evaluated at days 14, 28, and 42. Isotype-specific titers were evaluated at day 42. Mice were then sacrificed at day 42, and splenocytes were restimulated with 5 μg/mL of HbsAg for 48 h; then supernatant was assayed for (D) Th1-biasing cytokines and (E) Th2-biasing cytokines. Reproduced with permission from ref (61).

β-glucans are β1→3 and β1→6 linked glucose-based polysaccharides found in fungal cell walls and recognized by a variety of immune receptors including complement receptor 3 (CR3), Dectin-1 (CD369), and TLR2. In particular, Dectin-1 is a C-type lectin with specificity for β-glucans which, when bound, can induce a host of downstream immune responses including NF-κB activation, phagocytosis, and induction of epigenetic immune memory.^55−58^ Moreover, innate immune activation by Dectin-1 has been shown to induce Th1 differentiation and elicit robust cellular immune responses.^59^ On account of their accessibility, desirable immunogenic profile in the absence of toxicity, and ability to form nanoparticles in solution, formulations comprising β-glucans are desirable alternatives to conventional adjuvants and have shown potential for vaccine formulations.^55^ In an example highlighting the application of β-glucans for vaccination, Donadei et al. show that soluble β1→3 linked glucans conjugated to diptheria toxin induce robust antibody responses when administered intradermally.^60^ The key benefit to this system is that it allows targeting of skin-resident dendritic cells without forming granulomas, which are seen when depot adjuvants such as alum are administered intradermally or subcutaneously (alum must be used intramuscularly as a result of this effect). Alternatively, insoluble β-glucan particles can form antigen depots with greater immunogenic capacity than alum, as shown recently in work by Soares and co-workers using Curdlan β-glucan particles (GPs).^61^ In this study, a variety of carbohydrate-based GP formulations including Curdlan, Curdlan with chitosan, and chitosan (see Section 3.4) were formulated as nanoparticulate delivery vehicles for hepatitis B surface antigen (HBsAg). It was found that Curdlan GPs formulated without chitosan were best internalized. When employed in a vaccine formulation with HBsAg, the Curdlan GPs induced significant antibody titers and Th1-associated cytokines, suggesting that particulate formulation and Dectin-1 binding can facilitate antiviral immune responses and that β-glucans and chitosan do not synergize for immunotherapy (Figure 2). These results and others^62−65^ highlight the potential of β-glucans as adjuvants for vaccines and cancer immunotherapies. The development of facile syntheses or isolations of precisely defined β-glucan scaffolds with optimized solubility and pharmacokinetics remains a roadblock for clinical translation and an area for future exploration by polymer chemists.

### 3.4 Chitosan

Chitosan is a positively charged, β1→4 glucosamine-based polysaccharide derived from chitin, a biopolymer that affords structural rigidity in a variety of plants, animals, and fungi. Chitosan drives dendritic cell maturation and induce Type 1 interferon (IFN) responses through a variety of innate immune receptors.^66−68^ Perhaps most notably, a seminal report by Carroll and co-workers^68^ demonstrates that chitosan can activate the STING (stimulator of interferon genes) pathway (see Section 4.2) to trigger APC maturation, costimulatory molecule expression, and Th1-biased adaptive responses. Specifically, chitosan exposure results in mitochondrial stress and reactive oxygen species (ROS) production, leading to an increase in mitochondrial DNA in the cytosol. Ultimately, activation of the STING pathway leads to Type 1 IFN production and Th1-biased responses based on this elicited “danger” signal.^67,68^ In addition to STING activation, chitosan activates the NLRP3 inflammasome, binds various PRRs (including TLR2, TLR4, and MMR), and can form antigen depots to mediate adjuvanticity.^69−72^ While this polyvalent mode of activation creates challenges for mechanistic studies, translational works involving chitosan remain an active area of research. In particular, various experiments have shown that chitosan enhances the adjuvanticity of intranasal vaccines.^73−75^ On account of its positive charge, chitosan more effectively traverses the mucosal membrane to deliver a given antigen and stimulate innate immunity than conventional adjuvants. As a result, it is used both in stand-alone formulations and as a component of nanoparticles to enhance the efficacy of vaccines and other therapeutics.

### 3.5 Glycolipids and Saponins

Glycolipids and saponins are an additional class of PAMPs composed of carbohydrate–lipid conjugates that may be effective agents for use in next-generation vaccine adjuvants.^5,76^ While the adjuvanticity of glycolipids has been demonstrated to function through multiple classes of PRRs (as discussed in excellent reviews^5^), here we focus on saponin and α-galactosylceramide (α-GalCer) systems that target new classes of receptors for immunomodulation. Saponins are naturally occurring, amphiphilic, terpene-containing oligosaccharides that have been used since the 1970s to facilitate robust antigen uptake, balanced Th1/Th2 responses, and potent IgG titers through multiple PRRs.^37,77−80^ Notably, saponin extracts are used in approved adjuvant formulations, AS01_B_, AS01_E_, and Matrix-M, for respective vaccines against shingles, malaria, and SARS-CoV-2.^36,81−84^ Work from den Brok and co-workers demonstrates one mechanism by which saponin-based adjuvants function.^85^ The authors show that saponin-based adjuvanticity is based on lipid body formation in dendritic cells which enhanced cross-presentation to CD8^+^ T cells via the immunoproteasome. This result supports the use of saponin-based adjuvants in cancer vaccines where CD8^+^ T cell responses are highly desirable. In other mechanistic studies by Marty-Roix et al. and Welsby et al., saponin adjuvants were found to activate the NLRP3 inflammasome (see Section 4.3) via lysosomal rupture in a cathepsin B- and MyD88-dependent manner.^86,87^ While the role of NLRP3 inflammasome activation in supporting adaptive immunity is debated,^88−90^ release of proteolytic enzymes and lysosomal contents into the cytosol supports the use of saponin adjuvants to enhance cross-presentation to CD8^+^ T cells in adjuvant formulations. Key limitations in the use of saponin adjuvants are their systemic toxicity, complex bioavailability, and limited abundance in nature.^91^ These limitations motivate the development of novel, synthetic glycolipid polymer adjuvants that target the NLRP3 inflammasome.

In contrast to saponins, α-GalCer adjuvants are easily synthesized glycolipids which are also found in nature as a structural component of the marine sponge, *Agelas mauritianus*. α-GalCer has been shown to target the CD1d receptor on APCs to facilitate activation of invariant natural killer T cells (iNKT cells), which are a subset of T cells that bridge innate and adaptive immunity by providing rapid T-helper cytokine production (such as IFN-γ) without requiring a classical peptide antigen. While early works using α-GalCer were plagued by low binding affinity to CD1d, a screen of synthetic α-GalCer derivatives was recently conducted, and an analogue, 7DW8-5, with increased affinity was identified for use in vaccine and immunotherapeutic applications.^92^ Building upon this work, Feng and co-workers^93^ tested the efficacy of 7DW8-5 relative to a conventional adjuvant, alum, when formulated in a commercially available influenza H5N1 quadrivalent vaccine. The authors found that the 7DW8-5-containing vaccine induced antibody titers that were comparable to the conventional alum-based adjuvant but conferred improved survival after a lethal H5N1 challenge.^93^ Importantly, these results are backed by mechanistic studies demonstrating potent Th1- and Th2-mediated responses due to the activation of iNKT cells.^94^ Facilitating iNKT cell proliferation using α-GalCer and other CD1d ligands is a new and exciting avenue for vaccines and immunotherapies, and engineering newer and more sophisticated formulations to target CD1d in synergy with other innate immune receptors could result in new and better therapeutics with desired and controlled immune response profiles.

## 4 Synthetic Polymers with Innate Immunostimulatory Activity

### 4.1 Conceptual Overview

The prevailing theory of pattern recognition supposes that the immune system responds to pathogen- or danger-associated molecular patterns.^6^ While synthetic polymers (such as (meth)acrylamides and (meth)acrylates) have not been designed with the expressed goals of activating such systems, recent studies have highlighted that polymer coils retain physicochemical properties and/or structural motifs that can allow them to behave as danger signals and activate PRRs to induce an immune response. As PRR ligation is better understood at the molecular level and polymers of increasing complexity can be facilely prepared, rational design could be employed to prepare polymers that disrupt organelle homeostasis,^95^ interact with biological receptors,^96^ and induce innate immunostimulatory activity in a controlled manner. If achieved, synthetic (i.e., nonbiologically derived or inspired) polymers that bind endogenous PRRs (or otherwise activate innate immunity) would be advantageous over conventional PRR agonists on account of their relative low cost, high tunability, and facile compatibility with existing vaccine or immunotherapy formulations, making immunostimulatory polymers desirable for clinical translation. Likewise, given the broad domain space of polymer synthesis and the breadth of materials currently in preclinical testing, developing strategies to predict and test the immunostimulatory capacity of synthetic polymers for nonimmunostimulatory applications (such as drug delivery) is desirable for rapid and accurate early stage screening of therapeutics.

Two PRRs that are amenable to targeting by synthetic polymeric danger signals and which are highlighted in this Perspective are the stimulator of interferon gene (STING) and the NACHT-, LRR-, and PYD-domain containing protein 3 (NLRP3) receptor systems. While other PRRs are targeted by highly specific ligand–receptor systems, the STING and NLRP3 receptors are unique in that they respond to broader classes of molecular signals. As such, their activation can be induced by diverse stimuli and hold high potential for activation by nonbiological polymers. Understanding design principles for polymeric agonists of these receptors is critical for the screening of nontoxic biomaterials and the design of next-generation polymeric therapeutics.

### 4.2 Synthetic Polymers That Activate STING

STING is a PRR that is activated by cytosolic DNA to induce interferon production.^97^ Similar to other innate immune sensors, STING is activated via a two-step pathway. First, cyclic guanosine monophosphate–adenosine monophosphate (GMP-AMP) synthase (cGAS) becomes activated upon binding DNA and catalyzes formation of cyclic GMP-AMP dinucleotide (2′-3′ cGAMP). In turn, 2′-3′ cGAMP binds STING to induce a conformational change and condense into a macromolecular aggregate, allow ligation with TBK-1, and generate an interferon response.^98−100^ Other bacterial-derived cyclic dinucleotides (CDNs), such as cyclic di-GMP and di-AMP, can similarly bind STING and serve a role in pathogen recognition and are being explored as novel adjuvants.^99^ The development of STING agonist-based cancer vaccines is an active area of research for several reasons: STING (1) is expressed in most cell subsets, (2) is present in immunologically “cold” (immune cell deficient) tumors, (3) is compatible with checkpoint blockade therapies, and (4) can facilitate IFN-mediated CD8^+^ T cell responses which are critical for tumor destruction.^101,102^ Recently, synthetic non-nucleotide STING receptor agonists that mimic the structure of CDNs have been prepared and shown to afford robust antitumor activity, spurring multiple early stage clinical trials probing safety and efficacy (NCT04144140, NCT03843359, NCT04609579, and NCT04420884 at the time of submission).^103−105^ Given the unique mode of activation of STING, whereby a conformational change of the protein structure induces its condensation, polymers can be synthesized to target such changes and provide a novel therapeutic modality for the treatment of disease.

**Figure 3:**
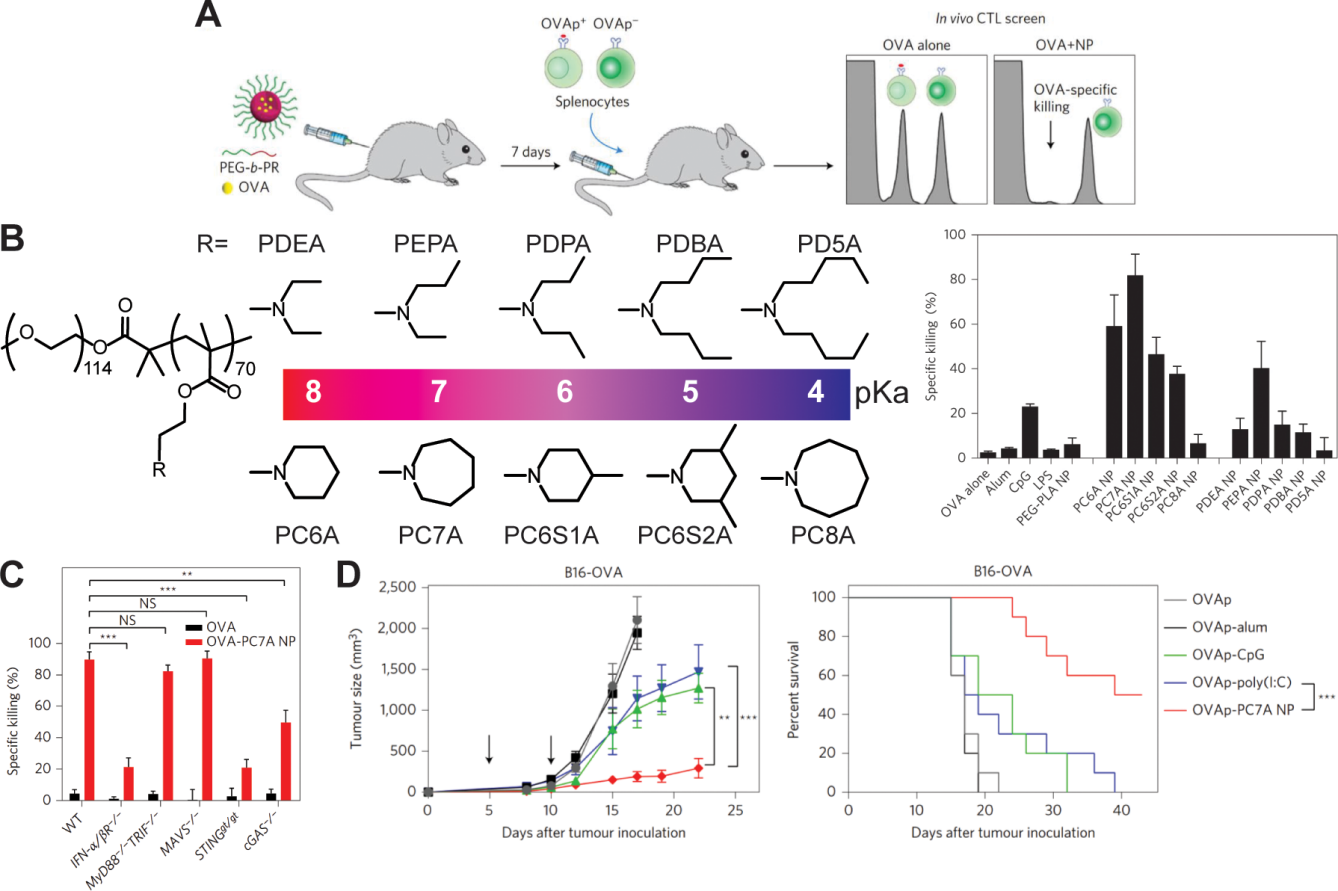
Synthesis and characterization of a STING-activating nanovaccine. (A) Schematic representation and screening method used to determine OVA-specific T cell-mediated killing of various cyclic amine nanoparticles loaded with OVA. (B) Efficacy of various cyclic amine nanoparticles or controls screened identified PC7A as an optimal adjuvant candidate. (C) Repeat of screening experiments in STING-, cGAS-, or IFN-α/β receptor-deficient mice identify a role of STING in PC7A nanovaccine efficacy. (D) Therapeutic vaccination with PC7A nanovaccine is shown to slow the growth of an aggressive B16-OVA melanoma. Reproduced with permission from ref (106). Copyright 2017 Springer Nature.

While 2′-3′ cGAMP and its analogues have been the focus of most small molecule STING agonist systems, recent work by the Gao laboratory has demonstrated that a synthetic block copolymer, poly[(ethylene glycol)-*b*-(2-(hexamethyleneimino)ethyl methacrylate)] (PC7A), can bind STING to induce downstream TBK-1 signaling and IFN production.^96,106,107^ By use of confocal microscopy, site-directed mutagenesis, and binding assays, it was shown that PC7A can access the cytosol and bind a noncompetitive site on both the mouse and human STING proteins. PC7A can therefore be used in combination with 2′-3′ cGAMP or other small molecule STING agonists for dual STING-targeted therapy, and the polymer was demonstrated to have robust efficacy for the treatment of multiple tumor models (Figure 3). This system is the first to our knowledge that employs a synthetic polymer to bind an endogenous ligand in a mechanistic fashion. The polymeric agonist has desirable properties when compared to synthetic STING ligands. It is prepared in a single step from low-cost, commercially available monomers, binds a noncompetitive STING surface site which allows its use in 2′-3′ cGAMP-resistant STING variants, forms nanoparticulate structures that facilitate enhanced cellular uptake relative to small molecule agonists, and can be tuned by variation of molecular weight or incorporation of dopant monomers. Additional screening of other cyclic amine-based methacrylates by the Gao group has shown that innate immune activation induced by PC7A is unique to the seven-membered ring structure (Figure 3),^106^ highlighting the specificity which will be required for future nonbiological polymeric agonists.

A further consideration in the targeting of STING for immunostimulatory applications is that any cargo must be delivered to the cytosol of target cells for effective therapy. Here, polymers can enhance stability and delivery of STING ligands, thereby increasing immunogenicity for their target application.^108^ Indeed, work by Shae and colleagues^109^ exploits the use of pH- and ROS-responsive endosomolytic polymersomes loaded with 2′-3′ cGAMP to target delivery of cargo to the tumor microenvironment.^109^ By injecting the polymersomes intratumorally or intravenously to mice carrying B16.F10 tumors, CD8^+^ T cell infiltration was enhanced 10-fold relative to 2′-3′ cGAMP alone. In combination with checkpoint blockade therapy, 4/10 mice treated intravenously with the polymersomes (but 0/10 treated with 2′-3′ cGAMP alone) completely cleared the tumors. In an alternative strategy, the negative charge of CDN STING agonists was leveraged to allow charged-complexed, polyvalent delivery using the Q11 peptide nanofiber platform. The Q11 nanofibers were functionalized with poly(ethylene glycol) (PEG) and a nona-arginine construct (PEG-Q11R9-CDN) to facilitate endosomolysis and subsequent STING activation.^110^ Using the PEG-Q11R9-CDN complexes, they could achieve selective delivery of the STING agonists to dendritic cells and subsequent activation in mice using a sublingual route of administration. Such advanced applications are advantageous for the coadministration of STING agonists with other PRRs to generate synergistic activation by a single construct.^6,111^ Similar strategies for the (co)delivery of STING agonists have been employed by others,^112−116^ and more advanced formulations are expected to emerge as the STING receptor is better understood.

### 4.3 Synthetic Polymers That Activate NLRP3

In contrast to STING, whose native ligands are specific to nucleotide agonists, the NLRP3 protein undergoes conformational change in response to a broader class of stimuli that behave as danger signals (DAMPs) after disruptions of homeostasis.^117^ While diverse stimuli have been implicated in NLRP3 activation, including reactive oxygen species,^118−120^ extracellular ATP,^121^ and lysosomal disruption,^122−124^ these stimuli likely converge on cellular potassium efflux as a causative agent of the NLRP3 conformational change.^117,125^ Once activated, NLRP3 can interact with ASC, NEK7, and pro-Casp1 to form the NLRP3 inflammasome, a megadalton protein complex with a host of effector functions.^117,126,127^ Specifically, NLRP3 inflammasome formation catalyzes cleavage of pro-Casp1 to Casp1. Active Casp1 then facilitates secretion of IL-1β and IL-18 and pyroptosis, a form of inflammatory cell death characterized by GSDMD N-terminal cleavage, cell membrane pore formation, and eventual cell lysis.^127,128^ While the NLRP3 inflammasome is implicated in pathologies including gout, Alzheimer’s disease, and septic shock, it has also gained attention as a potent PRR for use in novel vaccines and immunotherapies.^88,89,117^ Specifically, saponin adjuvants activate the NLRP3 inflammasome via lysosomal disruption as described in Section 3.5 to induce potent IL-1β signaling for initiation of an adaptive immune response.^86,87^ While effective, saponin-based systems are costly, derived from limited natural resources, synthetically complex, and prone to toxicity.^91^ Synthetic polymer-based alternatives that activate the NLRP3 inflammasome are desirable for use as adjuvants which overcome these limitations and allow broad applicability of this technology. Moreover, given the disease states and toxic side effects associated with inflammasome activation, developing a molecular level understanding of the physicochemical relationships between polymer–cell interactions, lysosomal disruption, inflammasome activation, and toxicity will be critical for the design of safe biomaterials.

**Figure 4:**
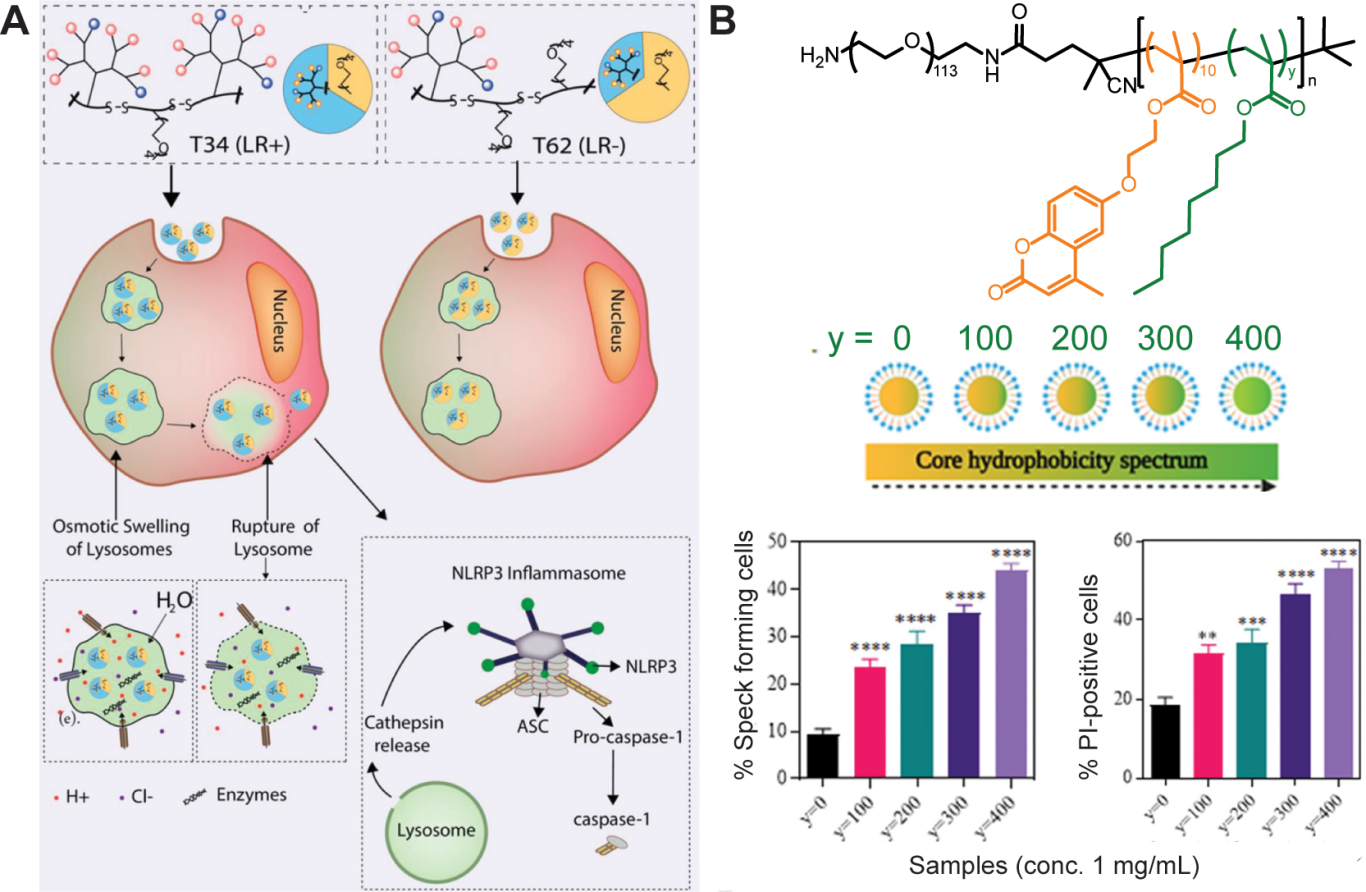
(A) Dendrimeric histidine- and tryptophan-containing scaffolds with 34 or 62% ethylene glycol (T34 and T62) were synthesized and shown to mediate NLRP3 inflammasome activation via a lysosomal rupture- and cathepsin-dependent mechanism. Reproduced with permission from ref (130). (B) Self-assembling polymer nanoparticles induce ASC speck formation and immunotoxic responses in a composition-dependent fashion, with increasing core octyl methacrylate content mediating maximal immunogenicity. Reproduced with permission from ref (131).

Recently, it was shown that cationic polymers can activate the NLRP3 inflammasome via endolysosomal disruption.^124,129−133^ Polymers are taken up by the cell and insert in the endosomal membrane catalyzing its rupture upon endolysosomal acidification. The rupture releases the lysosomal contents (including cathepsins) into the cytosol and mediates NLRP3 inflammasome activation.^130,131^ While the precise mechanism by which polymers disrupt the endolysosome to activate the NLRP3 inflammasome is incompletely understood, we and others have shown that the properties of polymers can modulate the extent of lysosomal rupture and provide an avenue through which controlled NLRP3 inflammasome activation can be achieved.^130−133^ Moreover, such lysosome-disrupting polymeric adjuvants can be formulated to deliver immunostimulatory cargo to the cytosol and activate cytosolic PRRs (such as STING) to afford multiadjuvant synergies. In a recent publication by the Esser-Kahn group, the composition of a dendrimeric scaffold composed of variable ratios of cationic amino acid and tetra(ethylene glycol) (TEG) domains was found to modulate the extent of osmotic swelling in the endolysosome following cellular uptake, thereby controlling the extent of rupture and the degree of downstream Casp1 and IL-1β activity (Figure 4A).^130^ Likewise, Baljon et al. report that the ratio of butyl methacrylate to 2-(dimethylamino)ethyl methacrylate in a pH-responsive copolymer could tune the extent of endolysosomal rupture and inflammasome activation in THP-1 cells,^132^ and Nandi et al. report that the alkyl content in poly[(ethylene glycol)-*b*-[(coumarin methacrylate)*-r-*(octyl methacrylate)] similarly influenced the extent of endolysosomal rupture and inflammasome activation in iBMDM cells (Figure 4B).^131^ These results highlight that subtle changes in physicochemical properties can have drastic impacts on endolysosomal rupture and provide rapid, high-throughput methods for the screening of NLRP3 inflammasome activation via IL-1β activity. Such screening is critical for the design of novel adjuvants and of polymeric biomaterial and drug delivery formulations. Future work must be conducted to generate *in vivo* correlates of these *in vitro* results to confirm translation to higher order systems.

Beyond this *in vitro* mechanistic work, synthetic NLRP3 inflammasome activating adjuvant constructs have been engineered to produce potent adaptive immune responses *in vivo*.^111,134−136^ Li et al.^135^ prepared poly(ethylenimine)-coated mesoporous silica rods complexed with CpG (a TLR9 agonist) and the APC maturation- and differentiation-supporting cytokine, GM-CSF. This formulation was found to generate significant innate immune activation marked by IL-1β secretion and induce antitumor immunity against multiple tumor lines in only a single dose.^135^ Providing a more mechanistic approach, the Takeoka group employed inflammasome-activating, arginine-containing liposomes loaded with a model antigen, ovalbumin (OVA), to probe antigen presentation and T cell activation.^133^ Here, it was shown that the ratio of cationic arginine groups to hydrophobic lipid tail influenced the extent of cellular uptake, endolysosomal rupture, and NLRP3 inflammasome activation. The liposomes which maximally rupture lysosomes were found to induce upregulation of cell surface activation markers, CD40 and CD86, and route antigens for presentation on MHC-I to facilitate a CD8^+^ cytotoxic T cell response.^133^ Other works have highlighted that IL-1β and IL-18 can synergize with IL-12 to invoke potent antitumor responses, providing an additional framework by which rationally designed adjuvant formulations based on inflammasome activation can be achieved.^137,138^ These works highlight the potential of inflammasome activation as a mediator of adaptive immunity, but future work must be conducted to better elucidate how polymer physicochemical properties and related inflammasome activation correlate with the *in vivo* response.^88−90^ Such structure–bioactivity relationships will allow the rational design of polymers for vaccines and immunotherapy.

## 5 Polymer–Drug Systems for Enhanced Adjuvanticity

### 5.1 Conceptual Overview

**Figure 5:**
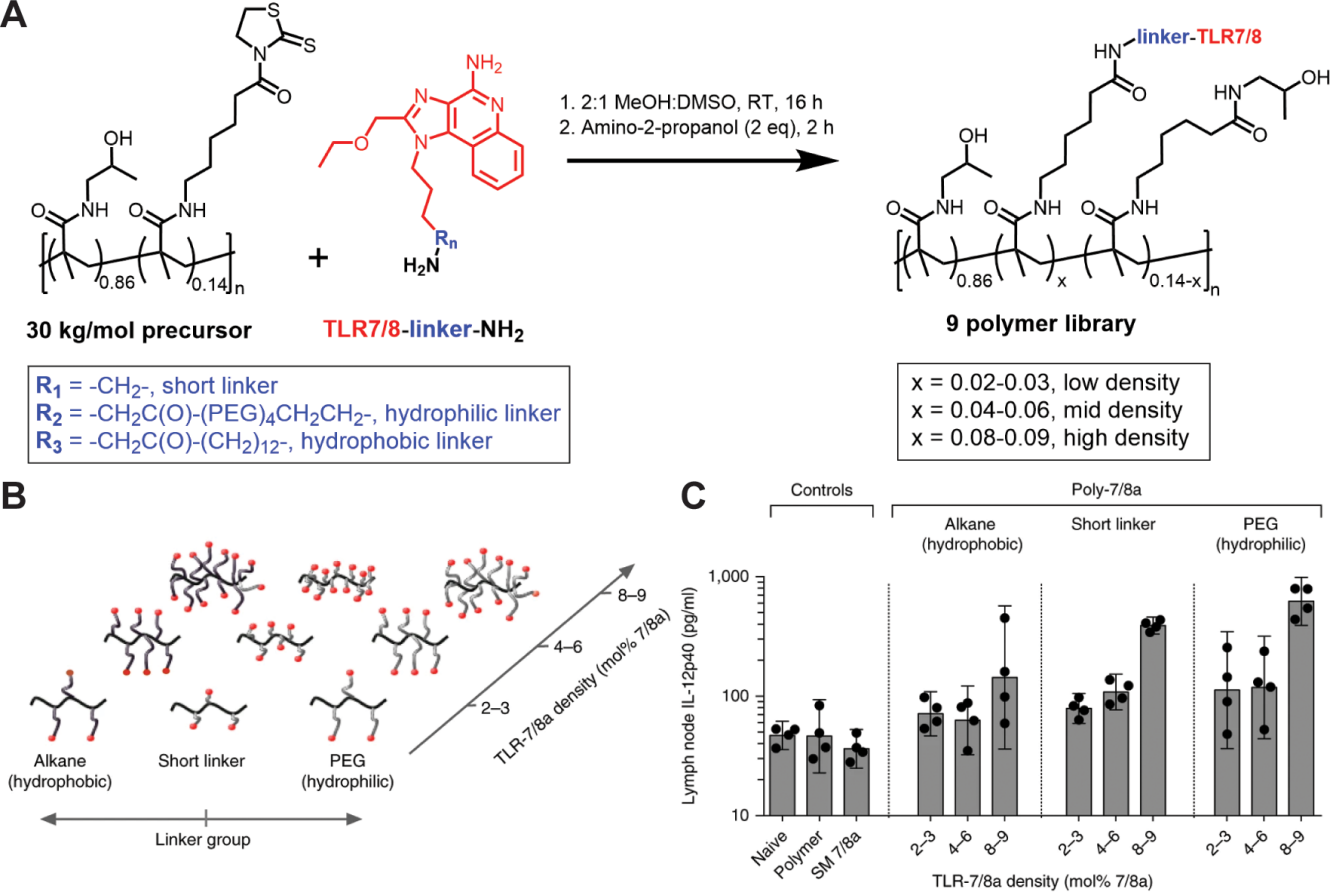
(A) Synthesis of a combinatorial polymer–TLR agonist library comprised by postpolymerization modification of a thiazolidine-containing, water-soluble polymeric scaffold. (B) Polymers with varied charge, TLR agonist density, and linker length were prepared. (C) IL-12p40 secreted by lymph node-derived cells 24 h after injection was then used to screen immunostimulatory activity of the polymers. Polymers that self-assemble into nanoparticles were found to maximize IL-12p40 secretion. Reproduced with permission from ref (7). Copyright 2015 Springer Nature.

Polymer–drug systems have gained attention as a method by which enhanced immunostimulatory activity can be achieved. Polymers covalently conjugated to or noncovalently assembled with immunogenic groups such as PRR agonists can allow for polyvalent receptor–ligand interactions, localized delivery of cargo, or delivery of multiple cargoes to a single locus. In immunology, such constructs have been employed in the design of novel adjuvants, which can concurrently deliver antigenic cargo and activate one or more PRRs for vaccination or cancer immunotherapy. Furthermore, it has been shown that physicochemical properties play a dramatic role in the resultant immunological activity of PRR–adjuvant conjugates or assemblies *in vivo.* This was demonstrated in a notable work by Lynn and Laga et al.^7^ where R848, a TLR7/8 agonist, was conjugated to an *N*-(2-hydroxypropyl)methacrylamide scaffold via a thiazolidine-2-thione reactive moiety and used as a vaccine adjuvant in nonhuman primates (Figure 5). In that work, nanoparticle formation, agonist density, and charge were all critical in mediating immunogenicity, providing a framework by which efficacious PRR agonist–polymer conjugates can be prepared.^7^ Given these results, we highlight physicochemical properties that can enhance or otherwise affect immune activity on account of the polymer backbone and then describe some covalent and noncovalent strategies that have been employed to generate highly immunogenic polymer–drug systems. While PRR agonist–polymer conjugates have been discussed previously because of their ability to facilitate immune synergies,^6,111,139,140^ we focus herein on the role of polymer properties and synthetic design in the innate activity of polymer–PRR agonist systems. Moreover, while not discussed herein, we note the critical importance of linker chemistry and degradation kinetics in the design of polymer–drug conjugate systems and direct the reader to excellent reviews on this topic.^141,142^

### 5.2 Physicochemical Parameters of Polymer–Drug Systems

The physicochemical properties of immunogenic cargo play a key role in their resulting immunostimulatory activity and bioavailability.^143^ Particle size can control routing of molecular cargo to the immune system; larger particles (>500 nm) form antigen depots at the injection site for processing by tissue-resident APCs, while smaller particles (<100 nm) quickly and directly drain to the lymph node and afford efficient presentation by lymph node-resident APCs.^144,145^ Unformulated soluble cargo (<10 nm), meanwhile, rapidly enters the bloodstream where it can induce off-target systemic side effects before being removed by the liver and/or kidneys.^8^ While the favorable pharmacokinetics of particulate systems are beneficial relative to those of soluble systems for the controlled delivery of adjuvants, both injection site- and lymph node-targeting strategies have found use in FDA-approved vaccines. This highlights the divergent strategies which can be employed in different contexts to develop a productive immune response.^37,146^ Despite the advantages of particulate systems, controlling size and precise physicochemical properties of such systems remains challenging and is further limited by poor encapsulation efficiency of chemically incompatible cargo (such as hydrophobic adjuvants). Next-generation polymeric vaccine systems will afford better control over the size of formulated materials and compatibility of materials incorporated therein to enhance stability and efficacy of the end-product.

Even among similarly sized nanoparticles, shape, charge, and texture can further modulate immune responses. The Mitragotri group and others have shown that particle shape plays a distinct role in phagocytosis and processing of antigen, with smaller, spherical particles (mimicking that of many natural pathogens) exhibiting maximum phagocytosis by APCs. In contrast, higher aspect ratio materials induce poor cellular uptake and cellular damage consistent with NLRP3 inflammasome activation and/or necrotic cell death.^147−150^ Charge can similarly modulate activity of polymer-containing immunogenic systems. Cationic polymers such as poly(ethylenimine) (PEI), poly(2-aminoethyl methacrylate) (AEMA), poly(*N*,*N*′-dimethylaminoethyl methacrylate) (DMAEMA), and polyarginine have been employed to complex negatively charged PRR agonists such as CpG (TLR9 agonist) or poly(I:C) (TLR3 agonist) and enhance uptake and cytosolic delivery.^110,112,135,151^ Such cationic polymers can effectively facilitate cytosolic delivery to enhance cross-presentation of antigen on MHC-I or to deliver mRNA and DNA for vaccination or gene therapy.^152−154^ While promising, cationic polymers often suffer from immunotoxic side effects on account of their ability to disrupt cellular or endolysosomal membranes, and further understanding of the relationship between physicochemical properties, immunogenicity, and toxicity of these materials in biological settings remains an active area of research.^153,155−158^ Lastly, the Kurt-Jones group has shown that particle texture can alter immune responses, with rough polystyrene nanoparticles inducing greater immunostimulatory activity and neutrophil infiltration than smooth particles.^159^ Collectively, these results highlight the many parameters which can be modulated in the design of an optimal immunotherapeutic agent.

### 5.3 Covalent Strategies to Develop Systems with Enhanced Adjuvanticity

**Figure 6:**
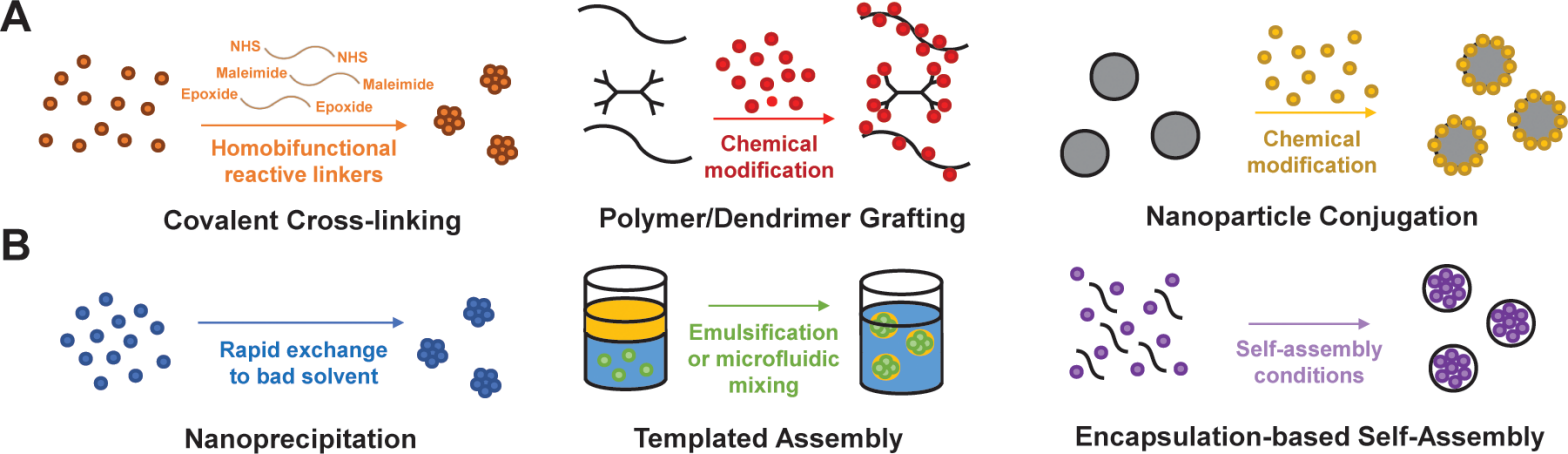
Overview of selected (A) covalent and (B) noncovalent strategies used to synthesize polymer-based vaccines or immunotherapies.

**Figure 7:**
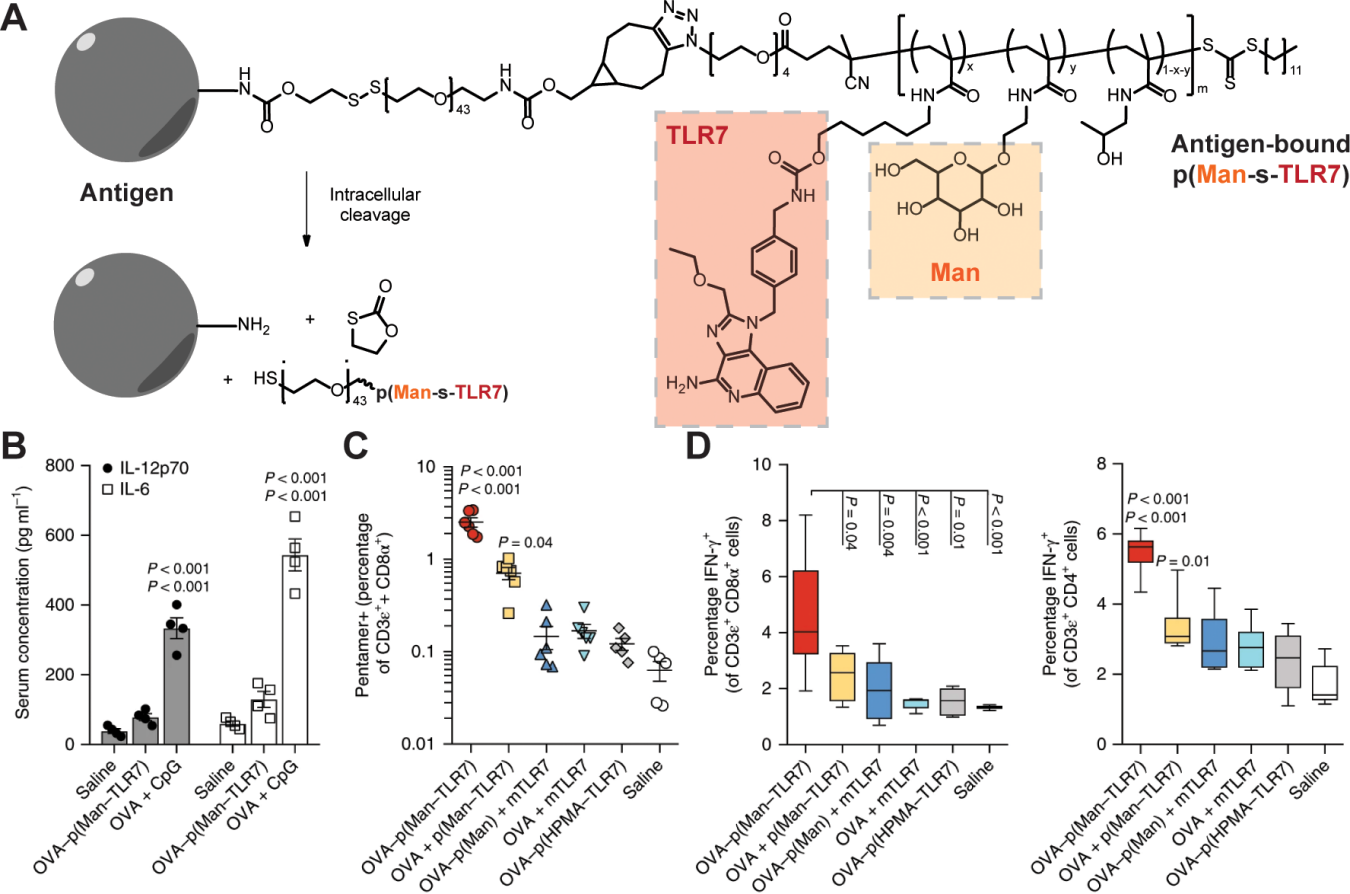
(A) Synthesis of p(Man-*s*-TLR7) glycoadjuvant containing a self-immolative disulfide linker to afford intracellular codelivery of antigen, mannose, and TLR7 agonist. (B) Vaccination with p(Man-*s*-TLR7) reduces systemic IL-6 and IL-12p70 production relative to soluble TLR9 agonist. (C) Antigen-specific CD8 T cell production as well as (D) antigen-specific CD8^+^ and CD4^+^ T cell activation after restimulation with the model antigen, OVA, were enhanced in the splenocytes of mice treated with p(Man-s-TLR7). Reproduced with permission from ref (51). Copyright 2019 Springer Nature.

Covalently linked combinations of synthetic polymers and additional immunostimulatory components are attractive for the design of adjuvants with precise molecular composition and behavior in solution. Several of the most attractive strategies for the design of covalent systems include linear polymers with PRR agonist grafts, dendrimers, functionalized solid nanoparticles (such as gold, silica, or poly(lactic-*co*-glycolic acid)), cross-linked hydrogels, and mechanically interlocked polymers (Figure 6A).^2^ The Hubbell group has demonstrated one application of polymer–drug conjugates to enhance the efficacy of vaccines.^51^ In this work, a TLR7 agonist-containing methacrylamide monomer based on the imidazoquinolinone class (pTLR7) and a mannose-containing methacrylamide monomer (pMan) were polymerized by using RAFT to obtain p(Man-*s*-TLR7). This polymer was conjugated to a model antigen, ovalbumin (OVA), by using a self-immolative disulfide-based linker to form an antigen-docked synthetic scaffold for immune activation (Figure 7).^51^*In vitro*, this platform show improved uptake and presentation of antigen using BMDC and T cell coculture experiments, and competition experiments using anti-CD206 and anti-CD209 antibodies (which block MMR and DC-SIGN, respectively) reveal that synthetic mannosylation was responsible for this response. After demonstrating the efficacy of this model by using OVA, p(Man-*s*-TLR7) was conjugated to the malaria circumsporozoite protein (CSP) and used in a murine vaccination study. Here, p(Man-*s*-TLR7) conjugated to CSP was shown to induce improved antigen specific T and B cell responses relative to unlinked controls. The Hubbell group has demonstrated in other works that this synthetic glycosylation strategy can be similarly employed by using GalNac and GlcNac as glycans to route antigens to the liver and induce tolerance in a model of diabetes.^52^ Glycans prepared by using the polymerizable monomer approach serve as an exciting area of exploration, and we await application for treatment of diverse disease states.

Beyond linear polymer scaffolds, dendrimers, functionalized nanoparticles, and cross-linked hydrogels can allow for higher density display of antigen or ligand as well-defined (and potentially stimulus-responsive) nanocarriers. Specifically, dendrimers can improve solubility and biocompatibility while displaying adjuvants at their surface on account of their globular structure.^160−163^ Wang and colleagues recently reported a light-responsive adjuvant therapy based on a TLR7-activating 2-aminoimidazole derivative and a poly(lysine) dendron. When complexed with an anticancer agent and antigen, dendrimeric light- and hypoxia-responsive nanoassemblies are formed which were found to display robust anticancer therapy against multiple tumor models.^164^ Cationic PAMAM-based systems have also been extensively employed for gene therapies and cancer therapeutics, but their toxic side effects have limited use in other applications such as vaccination or drug delivery.^158,165^ Similarly, polymeric or inorganic nanoparticles can be functionalized with PRR agonists to afford polyvalent display and reduce systemic side effects relative to soluble ligands. Such materials been extensively reviewed elsewhere with the chief limitation of this approach being that many solid materials are poorly biocompatible and biodegradable.^2,3,25^ Chemically cross-linked hydrogels, meanwhile, show great potential for generating immunogenic adjuvant systems with tailored physicochemical properties, biocompatibility, and release of synthetic or biologically derived cargoes.^166−172^ Demonstrating the potential of this synthetic approach, the Irvine lab has developed protein nanogel “backpacks”, which can be tethered to CAR T cells (engineered T cells with a scFv acting as a TCR) to support proliferation after adoptive cell transfer therapy.^167^ The backpacks contain recombinant IL-15, which supports T cells proliferation, and CD45, which serves as an anchor to the T cell surface, and are cross-linked at lysine residues by using a disulfide-containing NHS-ester linker.^167^ The backpacks were found to enhance T cell proliferation 16-fold relative to CAR T cells delivered with soluble IL-15, and this technology is now in Phase I clinical trials for the treatment of solid tumors (NCT03815682). While reversible bonds have found use in both chemistry and biology during the past decade, better strategies for the stimulus-responsive release of cross-linked biologicals under specific conditions will allow targeting of various immune cell subsets.

Finally, mechanically interlocked materials (such as polyrotaxanes and slide ring gels) can enhance avidity by allowing threaded ligands to freely move along a linear polymer axis.^173^ While basic proofs of concept have been demonstrated by using this approach,^174−176^ advances in the controlled synthesis of interlocked materials^173^ now allow the advantages of such materials to be realized for drug delivery and immunostimulatory polymer applications.

### 5.4 Noncovalent Strategies to Develop Systems with Enhanced Adjuvanticity

Spontaneous self-assembly or controlled nanoformulation of components using noncovalent strategies is an alternative strategy to achieve immunogenic materials. The advantages of such noncovalent strategies are that they are easily prepared from low-cost starting materials, break down on biologically relevant time scales into biocompatible byproducts, and can be imparted with stimuli responsive or other desirable properties.^167,177,178^ Formulations including imiquimod (a small molecule TLR7/8 agonist) serve as an example of the promise of formulated nanomaterials; while systemic toxicity after injection has prevented clinical translation of imiquimod as vaccine adjuvant, a lipid-modified derivative, 3M-052, adsorbed onto alum has shown remarkable safety and efficacy in preclinical studies and is now undergoing early stage clinical trials for prophylactic influenza vaccination when codelivered with antigen (NCT04177355).^179−181^ With these results in mind, we highlight the design of self-assembled materials as well as disordered nanoaggregates (Figure 6B) that hold potential for immunological applications.

Self-assembled delivery systems such as liposomes, lipid nanoparticles, micelles, and polymersomes have gained significant attention in the past decade. These systems are desirable because of their spontaneous self-assembly, synthetic reproducibility, high biocompatibility, quality of stabilizing reactive cargo, and ability to release material on biologically relevant time scales. On account of their amphiphilic properties, they also can encapsulate both hydrophilic and hydrophobic cargoes, making them highly versatile for the delivery of chemically diverse materials. While liposomes and lipid micelles have been extensively reviewed for drug delivery,^25,182^ perhaps the most notable recent application of self-assembled lipid-based nanocarriers for immunological applications has been in the delivery of mRNA. Here, lipid nanoparticles have been FDA-approved for vaccination against SARS-Cov-2 in 2021.^183^ In these systems, the ionizable lipid nanocarrier stabilizes mRNA from degradation and, upon endocytosis, assists in endosomal escape to deliver mRNA to the cytosol.^183,184^ mRNA plays a dual role in encoding for the production of antigen while also behaving as an adjuvant, acting on multiple PRRs including TLR3, TLR7, and RIG-I to stimulate a Th1-biased immune response.^183,185^ The lipid composition plays an important but poorly understood role in the resultant immune response and remains an active area of research.^183^ Alternative to lipid-based systems, polymersomes and polymeric micelles can be prepared that allow greater synthetic control over the molecular architecture and can confer stimuli-responsive behavior to the delivery system. Demonstrating these advantages, Dowling and Scott et al.^186^ synthesized a series of poly(ethylene glycol-*b*-propylene sulfide) polymersome-based vaccines loaded with a small-molecule TLR8 adjuvant and antigen. They compare the effects of different polymersome size and antigen loads on immunogenicity of the polymersome-based vaccines relative to live attenuated Bacillus Calmette–Guérin (BCG) vaccine.^186^ Maximum innate and adaptive immune responses are achieved with the polymersomes when size and antigen load are matched to the properties of the live attenuated virus, providing further design principles for next-generation therapies.

In contrast to self-assembled systems, chemically irregular noncovalent formulations can be achieved by nanoprecipitation or *in situ* hydrogel formation. Nanoprecipitation involves rapid transfer of cargo from a good solvent (often methanol or dimethyl sulfoxide) to a bad solvent (such as aqueous phosphate buffer) via dialysis or microfluidic mixing. As an example of this strategy, the Esser-Kahn lab has synthesized a poly(orthoester) scaffold which assembles by nanoprecipitation with a heterodimeric TLR2/6 and TLR7 agonist and antigen.^187^ When the resultant ∼50 nm constructs are administered as a cancer immunotherapy to mice bearing an aggressive B16.F10 melanoma, complete remission of the tumor is achieved.^187^ This formulation furthermore reduced systemic side effects relative to soluble TLR2/6 and TLR7 agonists, likely by prolonging bioavailability relative to the soluble formulation.^187,188^ Nanoprecipitation is a powerful approach to encapsulate large quantities of immunogenic materials and deliver them to specific cell subsets; however, it is limited by solvent compatibility of the cargo needed for successful nanoaggregate formation. Alternatively, the solvent compatibility requirement can be eliminated entirely by encapsulating cargo in hydrogels. In a recent example applied to vaccine delivery, the Appel lab has synthesized a polymer–nanoparticle hydrogel formulation composed of dodecyl-modified hydroxypropyl methylcellulose (C12-HPMC) loaded with PEG–PLA nanoparticles.^189^ This system is desirable because it can be formulated with both hydrophilic and hydrophobic cargo and injected through a syringe on account of its shear thinning behavior. The hydrogels, when formulated with a hydrophilic model protein antigen and a hydrophobic TLR3 agonist, displayed a depot effect at the injection site for more than 1 week and enhanced antibody responses 90 days after injection relative to a soluble formulation in the absence of a booster dose. Such delayed release formulations could enhance vaccine compliance and accessibility, but tuning the formulation to control release kinetics over relevant time scales remains challenging and an area of exploration.

## 6 Polymer–Drug Systems for Controlled Delivery of Cargo

### 6.1 Conceptual Overview

While polymers can enhance the adjuvanticity of immunostimulatory formulations as described in Section 5, they must also release cargo to specific cell subsets in the absence of immunotoxic side effects.^2,190^ To achieve this requirement, polymer–drug formulations can be imparted with stimuli responsive characteristics by using reversible chemistries or biodegradable linkages to allow release of molecular cargo under specific cellular or subcellular conditions, such as the reductive tumor microenvironment or acidic endolysosome. Alternatively, targeting ligands (often peptides that bind specific receptors) can allow delivery to specific cell subsets. Here, we discuss chemistry used in the design of several classes of responsive materials for immunological applications: pH-responsive materials for endolysosomal disruption, reactive oxygen species (ROS)-responsive materials for tumoral delivery, biodegradable polymers and peptides for slow release of cargo, and thermally responsive materials for delivery to metabolically active tissues. Furthermore, we discuss the incorporation of targeting peptides into the polymer backbone for the delivery of molecular cargo to specific cell subsets and/or organelles.

### 6.2 pH-Responsive Materials for Cytosolic Delivery

Various chemistries can be employed to prepare polymers that decompose or undergo physicochemical change in response to endolysosomal acidification to deliver cargo into the cytosol. Endolysosomes are cellular compartments in APCs that contain proteolytic enzymes and maintain a pH of 4–6. Upon APC activation, a decrease in endolysosomal pH can accelerate proteolytic processing and invoke antigen presentation on MHC-I and/or MHC-II. Such processing, in parallel with PRR signaling, is critical for the initiation of an adaptive immune response, making the endolysosomes of APCs attractive targets for the delivery of immunostimulatory cargo. To do so, pH-responsive chemistry can be employed. Some examples of responsive groups include (1) acetal- or hydrazone-based linkers that break and alter polymer morphology upon cleavage,^134,191^ (2) amine-, carboxylate-, or imidazole-containing polymers that undergo an acid/base transition at biologically relevant p*K*_a_ values,^109,116,132,153,157,192^ and (3) reversible charge complexes that decompose under particular conditions.^193−195^ For vaccine and cancer immunotherapy development, pH-responsive materials can be combined with immunostimulatory ligands to create nanostructures that can target endosomal or cytosolic immune receptors. Highlighting a creative application of this strategy, Gong et al. reported a pH-responsive copolymer that undergoes conformational change from 100 nm spherical structures to 5–8 μm nanosheets and delivers cargo upon endolysosomal acidification.^134^ These polymeric assemblies mechanically rupture the lysosome to activate the NLRP3 inflammasome and deliver antigen to the cytosol to facilitate antigen presentation on MHC-I. This “nanotransformer” vaccine was found to induce potent CD8^+^ T cell responses and facilitate complete B16.F10 tumor regression in combination with checkpoint blockade therapy in mice.^134^ These results highlight the interplay between polymer engineering and immune recognition and inspires design principles for future polymer adjuvant applications.

### 6.3 Using ROS as a Trigger for Tumoral Delivery

Reactive oxygen species (ROS) are a byproduct of metabolically active cells and are thus produced at high levels by rapidly proliferating cancer cells in the tumor environment.^196^ Given the chemical reactivity of oxygen radicals with functional groups (for example, in the reduction of disulfide/diselenide, arylboronic ester, or aminoacrylate bonds), ROS production can serve as a selective trigger for the degradation of polymers and/or the site-specific delivery of cargo to the tumor microenvironment. Synthesis of ROS-responsive polymers has been expertly reviewed.^197^ For cancer immunotherapy, such selective triggers can be used to deliver otherwise toxic doses of immunogenic material to the tumor site and facilitate otherwise inaccessible levels of cytotoxic T cell infiltration. Liang et al. recently published^112^ an immunotherapeutic system composed of an anticancer agent, SN38, functionalized with a reducible disulfide linker and a methacrylate handle. The resultant SN38 monomer construct was incorporated within a cationic triblock copolymer scaffold (poly(ethylene glycol-*b*-SN38 methacrylate-*b*-diethylaminoethyl methacrylate)) and subsequently self-assembled with DMXAA, a small molecule STING agonist specific to mice, to form 30 nm particles (pSN38-STING). In the reductive tumor microenvironment, the disulfide bonds are cleaved, and the pSN38-STING scaffold disassembles to trigger release of both DMXAA and SN38. The pSN38-STING particles were used as a therapeutic in a B16.F10 tumor model, where it was shown that they induced complete regression of an aggressive melanoma when administered with checkpoint blockade.^112^ As demonstrated in this work and others, ROS-responsive nanoformulations are often combined with anti-PD-1 or anti-CTLA4 checkpoint blockade or cytokine-based therapies to further enhance T cell activity.^112,167^ Moreover, delivery systems with combined ROS- and pH-responsive properties can direct delivery of immunogenic cargo to antigen-presenting cells in the tumor microenvironment.^198−202^ Such multi-stimuli-responsive systems offer the promise to be instrumental in developing advanced therapeutics that afford clinical efficacy without toxicity.

### 6.4 Thermally Responsive Materials for Controlled Release

Thermally responsive, synthetic materials provide a facile approach to drug encapsulation and release to metabolically active sites (such as the site of an infection or the tumor microenvironment). Polymers can exhibit either a lower or upper critical solution temperature (LCST or UCST), at which point the solubility of material in its aqueous environment is reversed.^203,204^ In LCST polymers, warming past the critical temperature induces a hydrophilic to hydrophobic transition. In this context, *N*-isopropylacrylamide (NIPAAm) has been studied extensively due to its LCST in aqueous solution (32 °C) near body temperature (37 °C). Early work by the Discher group demonstrates the application of NIPAAm-based vesicles for payload delivery upon application of a temperature stimulus.^205^ By designing amphiphilic diblock copolymers composed of NIPAAm and ethylene glycol, these vesicles could self-assemble and maintain their morphology upon injection. Applying a local cold pack resulted in disruption of structure to deliver cargo at a target site of interest. This technique is envisioned as a tool for chemotherapeutic delivery of toxic agents selectively to tumors. In an alternative strategy, Nishimura and co-workers probed the temperature-induced release of macromolecular payloads from maltopentose-*b*-poly(propylene oxide) vesicles.^206^ Using small-angle X-ray scattering and confocal microscopy, it was demonstrated that the formed vesicles dissociated in a multistep fashion upon cooling to 0 °C. It should be noted, however, that only ∼1–2% loading efficiency was demonstrated for their macromolecular payloads. Low loading efficiencies and poor control over the kinetics of payload release have hindered application of these techniques, and better synthetic strategies are needed for clinically relevant translation.

### 6.5 Biodegradable Polymers and Peptides for Controlled Release

For effective clinical translation of polymer-based drug delivery and adjuvant systems, the polymeric carrier must degrade on a clinically relevant time scale or otherwise avoid immunotoxic side reactivity and foreign body responses. To achieve this goal, a common approach is to employ biodegradable polymers such as poly(lactic-*co*-glycolic acid) (PLGA), poly(hydroxybutyrate), poly(β-amino esters), or naturally occurring carbohydrates, which are hydrolyzed under biological conditions to naturally occurring small molecules.^2,17,207−209^ Such biodegradable systems are dually advantageous because, in addition to their lower immunotoxicity, they can be designed to release their payloads over kinetically controlled time scales. To this end, PLGA nano- and microparticles have been extensively studied as slow-release vaccine delivery systems.^209^ Such particles can be synthesized with controlled size properties by using emulsion polymerization to facilitate lymphatic delivery^145^ and simultaneously co-encapsulate small-molecule PRR agonists and subunit antigens; more information regarding PLGA-based drug delivery can be found in excellent reviews.^209,210^ In addition to synthetic biodegradable polymers, peptide-based drug delivery can be advantageous on account of their biocompatibility, high degree of synthetic tunability, and ability to incorporate non-native functionalities.^110,211−213^ Peptides can be designed to prolong bioavailability or rapidly degrade on account of their susceptibility to react with endogenous proteases,^214^ and they can further be employed to deliver cargo to specific (sub)cellular compartments as described in Section 6.6. The toxicity or immunogenicity of peptide linkers can be modulated by modifying amino acid composition, and screening can be accelerated by recursive bio-based screening methods such as phage display.^211,215,216^ For example, Kang and colleagues used phage display to identify a nontoxic peptide that improves trafficking of macromolecules across the intestinal mucosal barrier for oral drug delivery.^216^ One key limitation in the synthesis of peptide-based systems is scalability relative to polymer synthesis or bacterial protein expression, as solid-phase peptide synthesis is costly, requires large quantities of toxic solvent, and is limited in the cases of difficult amino acid sequences or self-assembled sequences.

### 6.6 Targeting Peptides or Ligands for Delivery to (Sub)cellular Compartments

Polymeric and nanoparticulate delivery systems can be localized to specific (sub)cellular compartments by using targeting ligands, most often peptides containing one or more of many well-defined localization sequences.^3,217−219^ To design such targeting peptides, well-defined chemistries that allow for materials with tailored physicochemical properties (including molecular weight, dispersity, and nanostructure), controlled peptide incorporation, and the ability to deliver additional immunogenic moieties within a synthetic polymer scaffold must be developed. The Gianneschi group has developed pioneering chemistries to achieve these goals. In an earlier work, ring-opening metathesis polymerization of peptide-functionalized norbornene-based monomers was used to synthesize high-density peptide brush polymers.^220,221^ By use of cell-penetrating peptides as a model system, it is shown that the synthesized peptide–polymer conjugates can resist proteolysis relative to unconjugated peptides and effectively deliver cargo to the cytosol in the absence of toxicity. More recently, the same group synthesized high-density peptide brush polymers using PET-RAFT polymerization with acrylamide-modified peptides in water or DMSO.^212,213^ By extending the high-density peptide brush polymers with a second, hydrophobic block, micellar nanoparticles that display the peptide brushes on their surface could be generated. This strategy is advantageous because it lacks postpolymerization modification or harsh conditions which can introduce toxic contaminants or heterogeneity into the system. Alternatively, polymers bearing amines, thiols, or alkynes can be modified by using click chemistry to engraft targeting ligands which may not survive radical polymerization.^3^ These approaches provide methods by which polymer–drug systems can be used to deliver cargoes to (sub)cellular compartments, preventing systemic toxicity while enhancing efficacy.

## 7 Conclusions and Future Directions

In this Perspective, we outline current strategies for the preparation of both biological and synthetic immunostimulatory polymers that target a broad range of receptors including C-type lectins, Dectin-1, STING, NLRP3, and TLRs. We then explore strategies, such as covalent or noncovalent combinations of polymers and PRR agonists and responsive or targeted delivery, to enhance the immunogenicity of vaccine and immunotherapeutic formulations. Recent advances in living polymerization, polymer–drug systems, and understanding of immunology have allowed encouraging increases in the rate of development for new therapeutic strategies. While drug delivery systems and responsive materials have been heavily explored in the past decade, biologically derived and synthetic polymers with innate immunostimulatory capacity have been unlocked by these advances and now comprise a novel field ripe for exploration. We now discuss areas that we find promising for the identification of new immunostimulatory polymers and strategies for rapid, low-cost, and effective biocompatibility screening.

### 7.1 Novel Receptors

While development of polymers that target common innate immune receptors, such as TLRs, has been well studied, there remains an opportunity to target novel receptors that confer different and desirable immunologic responses. Recent advances in carbohydrate chemistry offer exciting opportunities to access novel polymers that target previously inaccessible lectins. For example, the Bertozzi group recently reported a strategy where *N*-carboxyanhydride polymerization is employed to target Dectin-1 or Siglec receptors.^222,223^ An advantage of such synthetic strategies is that additional functionality can be built into the polymeric agonists to develop materials that can target multiple receptors or deliver various components. In addition to the promise of biologically active carbohydrate-based polymers, recent advances in immunology have identified new receptors that can be targeted for immunotherapy. Notably, the DNGR-1 receptor (also called CLEC9A or CD370) was recently found to bind F-actin–myosin and confer cross-presentation of phagocytosed antigens on MHC-I to direct CD8^+^ T cell responses.^224^ Given the polymeric fiber-like structure of F-actin–myosin and desirability of CD8^+^ T cell-directed responses in cancer immunotherapy, synthetic DNGR-1 agonists are an attractive area for future study. Developing strategies to synthesize polymeric agonists of these receptors and target appropriate cell subsets with specificity will result in novel applications of biomaterials.

### 7.2 High-Throughput Synthesis

Beyond targeting novel receptors, developing polymers with better binding affinity and avidity or that can disrupt organelle homeostasis to activate innate immunity is an attractive strategy for the design of next-generation therapeutics. A growing body of work from the Esser-Kahn lab and others^130−132^ indicates that small differences to polymer physicochemical properties can have large impacts on immunostimulatory activity of synthetic polymers targeting the NLRP3 inflammasome. As such, better methods for synthesis, characterization, and screening of the immunostimulatory activity of polymers targeting both the NLRP3 inflammasome and other immune receptors will allow structure–bioactivity relationships to be developed over a larger domain space. Advances in high-throughput polymer synthesis make such screens possible.^225−227^ In a strategy pioneered by the Gormley group, polymer–drug conjugates were synthesized in an oxygen tolerant, one-pot approach using PET-RAFT in DMSO. Pendant cyclopropenone-protected cyclooctynes were then functionalized with azide-modified peptides or ligands under ambient conditions and purified via size exclusion chromatography to generate libraries of ligand–polymer conjugates for screening in 96- or 384-well format.^227^ This approach is low cost as well as scalable and provides a high degree of synthetic control in terms of monomer selection, polymer chemistry, and postpolymerization modification. These characteristics make such approaches ideally suited for translation. An example of the promise of high-throughput polymer synthesis in drug candidate screening was recently reported by the Appel lab, where a screen of 90 polymer-functionalized insulins was used to develop an ultrafast-acting insulin formulation with greater stability and efficacy in a porcine model of diabetes.^228^ Here, polymer composition was shown to alter the biodistribution, pharmacokinetics, and activity, highlighting the impact that polymer design can have on downstream applications.

### 7.3 Computation-Guided Discovery

In tandem with high-throughput screening, machine learning and computational prediction will further direct and accelerate the discovery of novel polymers with desirable immunological properties. Machine learning will allow emergent trends in functional polymers to emerge, as was recently discussed by Gormley and Webb,^229^ while further computational strategies can be employed to model polymeric interactions with biological target receptors such as PRRs or cancer proteins. The discovery of a STING-activating polymer^96,106,107^ by the Gao laboratory highlights the potential of synthetic immunostimulatory polymers (Figure 2), yet to unlock the full potential of this approach, polymers that can better recapitulate the enormous structural complexity of biological systems must be developed. To achieve this goal, strategies applied from the prediction of protein structure and ligand–receptor binding can be applied.^230−232^ Demonstrating mechanisms necessary to achieve this goal, work by the Baker group has identified that computational prediction can be employed to synthesize biopolymers that assemble in precise three-dimensional topologies,^233−236^ and such strategies were used to generate a peptide vaccine that mimics the structure of natural virus to afford potent B and T cell responses in a respiratory syncytial virus (RSV) model.^236^ Iterative strategies combining machine learning and high-throughput screening (i.e., machine-learning-guided directed evolution)^237,238^ can be used to direct discovery of polymers that can bind innate immune receptors or achieve well-controlled adaptive immune responses. Achieving analogous structural complexity in synthetic polymer scaffolds and directing assembly to form structures that can bind with immune receptors is an exciting frontier which is on the forefront of possibility in the next decade.

### 7.4 Biocompatibility Screening: A Double-Edged Sword

**Figure 8:**
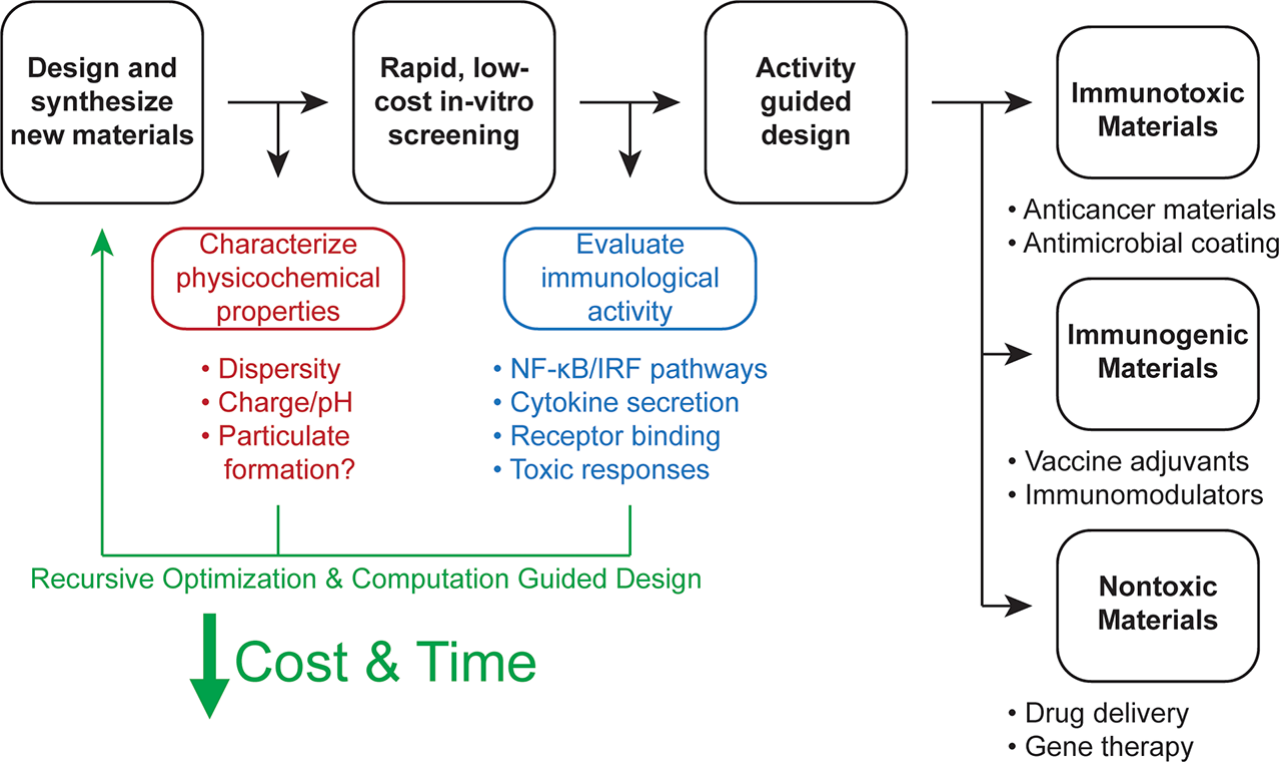
Proposed strategy for the low-cost and high-throughput screening and optimization of biomaterials for immunogenic and drug delivery applications.

As noted throughout this Perspective, the relationship between biomaterial efficacy and toxicity is inextricably linked. Many of the properties such as protein–polymer interactions, membrane disruption, or reversible chemistries that can make polymeric materials effective as adjuvants can also induce cell necrosis, complement activation, and toxic tissue accumulation in different contexts.^190^ Moreover, the repetitive structure of polymers, especially PEG-based drug delivery systems, have been shown to induce antipolymer antibodies, resulting in rapid clearance of biologically active cargo and undesired hypersensitivity reactions.^239^ Given this double-edged sword of efficacy and toxicity, developing rapid, translatable, and consistent methods to screen the immunological activity of polymeric materials at the preclinical development stage is critical to avoid costly translational research of toxic biomaterials. Currently, a lack of streamlined characterization tools has led to a scattershot of poorly defined *in vitro* and *in vivo* assays and has hindered progress in this domain. As such, in the screening of new materials for immunomodulatory and biomedical applications, we propose a series of simple and inexpensive *in vitro* experiments that can rapidly provide immunological information about novel immunomodulatory polymers. Such experiments were developed in the Esser-Kahn group for screening polymeric drug delivery systems and have proven broadly effective in rapidly predicting the *in vivo* safety and efficacy of broad classes of water-soluble or -dispersible polymeric materials (Figure 8).^240−243^

To test the immunological activity of polymeric materials, it must first be confirmed that the materials are free of endotoxin contamination derived from the synthesis or purification process. While the gel clot *Limulus* amoebocyte lysate (LAL) assay is the most common test for endotoxin contamination, this test relies on clotting of the coagulogen protein upon binding of endotoxins. As polymers often induce nonspecific clotting upon interaction with coagulogen, alternative tests are preferred.^243,244^ We have found that incubating polymers with the HEK TLR4 reporter cell line can provide a rapid and accurate readout of endotoxin contamination. In the case of endotoxin contamination, depyrogenation (i.e., removing endotoxin) can be achieved by heating, acid treatment, or extraction of endotoxic contaminants to achieve “clean” materials for preliminary testing.^244^ After endotoxin removal, *in vitro* immunostimulatory capacity can be assayed to determine the use case for polymers of interest. While several assays for immunological compatibility are employed in the literature, we and others have found cellular toxicity, NF-κB and IFN signal transduction pathway activation, and IL-1β secretion serve as useful predictors for polymeric materials.^240,241,243,245^ NF-κB and IFN gene expression are critical markers of early innate immune activation and, by using genetically encoded reporter cell lines, can serve as a low-cost alternative to multiplexed cytokine panels.^6,246,247^ IL-1β secretion and toxicity provide further information about immunotoxic cell death and can be rapidly assayed *in vitro* by quantifying secreted analytes with colorimetric assays such as ELISA. These tests can be conducted in both secondary and primary cells, although lack of reporter genes in primary cells require more laborious cytokine analysis (such as cytokine bead arrays^248^) to provide immunostimulatory information. Applying this early stage immunological compatibility testing in parallel with high-throughput synthesis and computation guided discovery will accelerate the screening and development of new polymers for immunomodulatory applications.
